# Efn1 and Efn2 are extracellular 5'-nucleotidases induced during the fission yeast response to phosphate starvation

**DOI:** 10.1128/mbio.02992-24

**Published:** 2024-12-11

**Authors:** Aleksei Innokentev, Ana M. Sanchez, Mara Monetti, Beate Schwer, Stewart Shuman

**Affiliations:** 1 Molecular Biology Program, Memorial Sloan Kettering Cancer Center, New York, New York, USA; 2 Gerstner Sloan Kettering Graduate School of Biomedical Sciences, New York, New York, USA; 3 Proteomics Core Laboratory, Memorial Sloan Kettering Cancer Center, New York, New York, USA; 4 Department of Microbiology and Immunology, Weill Cornell Medical College, New York, New York, USA; Harvard Medical School, Boston, Massachusetts, USA

**Keywords:** *Schizosaccharomyces pombe*, phosphate starvation, phosphate scavenging, 5'-nucleotidase

## Abstract

The fission yeast *PHO* regulon genes *pho1*, *pho84*, and *tgp1*—encoding a cell surface-associated acid phosphatase (Pho1), a plasma membrane inorganic phosphate transporter (Pho84), and a plasma membrane glycerophosphocholine transporter (Tgp1)—are strongly upregulated in response to acute phosphate starvation, as are the *SPBPB2B2.06c* and *SPAC1039.02* genes that encode putative 5'-nucleotidase paralogs of the binuclear metallophosphoesterase enzyme superfamily. Via proteomic analysis of the medium harvested from phosphate-replete and phosphate-starved fission yeast, we define a starvation secretome that includes SPBPB2B2.06c (renamed Efn1, for **e**xtracellular **f**ive-prime **n**ucleotidase), SPAC1039.02 (henceforth Efn2), and Pho1 among the most abundant exported proteins elaborated by phosphate-starved cells. We demonstrate and characterize a 5'-nucleotidase activity secreted into the medium of phosphate-starved *efn1*^+^*efn2*^+^ cells, which is eliminated by simultaneous deletion of *efn1* and *efn2*. By singly deleting *efn1* and *efn2*, we find that Efn1 contributes the greater share of secreted 5'-nucleotidase activity. Efn1 and Efn2 catalyze the release of inorganic phosphate from all four standard ribonucleoside monophosphates, in order of preference: CMP > UMP > AMP > GMP. Whereas *efn1*^+^*efn2*^+^ cells can use extracellular CMP as a source of phosphate during phosphate starvation, *efn1*∆ *efn2*∆ cells cannot. The secretion of 5'-nucleotidase enzymes during phosphate limitation is a newly appreciated facet of fission yeast phosphate homeostasis.

**IMPORTANCE:**

*Schizosaccharomyces pombe* adapts to phosphate starvation by upregulating the expression of a cell surface acid phosphatase that mobilizes inorganic phosphate from the extracellular milieu, as well as transmembrane transporters that take up inorganic phosphate and glycerophosphocholine. This study identifies two paralogous extracellular 5'-nucleotidase enzymes, Efn1 and Efn2, encoded by genes that are highly transcriptionally induced during acute phosphate starvation, as major proteins secreted into the medium by phosphate-starved fission yeast cells. Secreted Efn1 and Efn2 catalyze the release of inorganic phosphate from all ribonucleoside monophosphates, with a preference for CMP. Secretion of Efn1 and Efn2 enables phosphate-starved fission yeast to thrive by using extracellular CMP as a source of inorganic phosphate. The starvation-induced production of extracellular 5'-nucleotidases adds a new layer of pro-adaptive function during phosphate limitation.

## INTRODUCTION

Cells from all domains of life respond to phosphate starvation by inducing the transcription of phosphate acquisition genes encoding (i) secreted or cell-surface-associated enzymes that mobilize phosphate from the extracellular environment and (ii) transmembrane transporters of inorganic phosphate or simple phosphate-containing compounds. In the fission yeast *Schizosaccharomyces pombe*, three genes comprising a phosphate acquisition (*PHO*) regulon*—pho1* (cell surface acid phosphatase), *pho84* (inorganic phosphate transporter), and *tgp1* (glycerophosphodiester transporter)—are repressed under phosphate-replete conditions by upstream lncRNA-mediated transcriptional interference and de-repressed during acute phosphate starvation when interfering lncRNA synthesis wanes (1–3). Induction of the *PHO* regulon during acute phosphate starvation depends on the transcription factor Pho7 that binds to target DNA sequences in the *PHO* mRNA promoters (4–8).

The *PHO* proteins abet phosphate acquisition as follows. Pho1 is a histidine acid phosphatase enzyme that hydrolyzes phosphomonoesters via a covalent enzyme-(histidinyl)-phosphate intermediate. The increase in Pho1 expression on the cell surface during phosphate starvation is easily quantified by assaying whole cells for conversion of *p*-nitrophenylphosphate to the chromogenic product *p*-nitrophenol (1, 3, 4, 9). Inorganic phosphate liberated by Pho1 is a high-affinity substrate for transport across the plasma membrane by Pho84, a member of the major facilitator superfamily (MFS) (10). Tgp1 is an MFS-family transmembrane transporter of the phosphodiester compound glycerophosphocholine (11).

Transcriptome profiling (RNA-seq) of fission yeast cells at sequential times over 2 days of phosphate starvation revealed that the three *PHO* regulon mRNAs*—pho1*, *tgp1*, and *pho84*—were upregulated by 300-fold, 50-fold, and 35-fold, respectively, in starved cells (3). Two other genes*—SPBPB2B2.06c* and *SPAC1039.02*—encoding putative extracellular 5'-nucleotidase paralogs with potential roles in extracellular phosphate mobilization were also rapidly and highly upregulated during phosphate starvation (3). The *SPBPB2B2.06c* gene is expressed at a very low level in phosphate-replete cells and its mRNA is increased by 28,500-fold at 12 h of phosphate starvation (3). *SPBPB2B2.06c* is the most highly upregulated mRNA in phosphate-starved cells in terms of fold induction. The *SPAC1039.02* mRNA is increased by 150-fold at 12 h of phosphate starvation (3).

**FIG 1:**
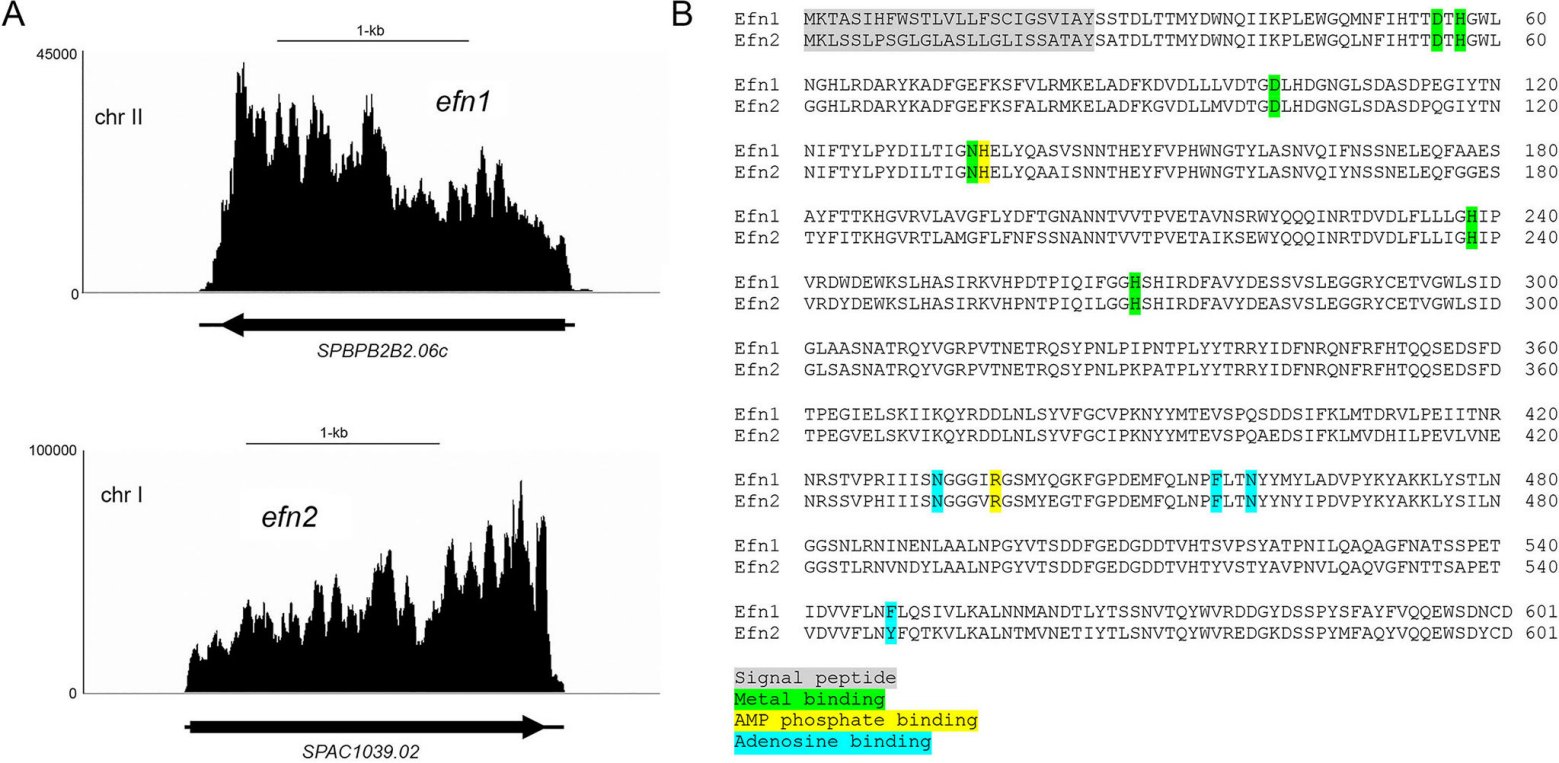
Predicted extracellular 5'-nucleotidases Efn1 and Efn2. (**A**) Strand-specific RNA-seq read densities (counts/base/million) are plotted as a function of position across the *efn1* and *efn2* gene loci in fission yeast cells after 12 h of phosphate starvation (data from reference 3). The x-axis scales are denoted by the 1 kb bars. The RNA-seq read densities were determined from cumulative counts of three RNA-seq replicates. The mRNAs are depicted as black arrows in the direction of their synthesis; the open-reading frames are denoted by thick lines, and the 5’ and 3’ UTRs are indicated by thin lines. (**B**) Alignment of the amino acid sequences of Efn1 and Efn2. Predicted active site constituents and an N-terminal signal peptide are highlighted in colored shading as specified below the alignment.

Virtually nothing is known about the fission yeast SPBPB2B2.06c and SPAC1039.02 proteins beyond their primary structures derived from the nucleotide sequences of their open reading frames (ORFs), located on chromosomes II and I, respectively (Fig. 1A). The distributions of RNA-seq reads across the *SPBPB2B2.06c* and *SPAC1039.02* genomic loci in phosphate-starved cells indicates that their mRNAs are not spliced and the ORFs are flanked by short 5' and 3' UTRs (Fig. 1A). SPBPB2B2.06c and SPAC1039.02 are 601-aa polypeptides that belong to the 5'-nucleotidase branch of the binuclear metallophosphoesterase enzyme superfamily. 5'-Nucleotidases, which catalyze hydrolysis of AMP to adenosine and inorganic phosphate, have been studied biochemically and structurally from bacterial and eukaryal taxa (12–15). 5'-Nucleotidases consist of an N-terminal metallophosphoesterase domain that binds the two metal cofactors and a C-terminal domain that engages the nucleoside monophosphate (NMP) substrate. Because the defining active site amino acids responsible for metal binding, AMP phosphate binding, and adenosine binding are conserved in the fission yeast SPBPB2B2.06c and SPAC1039.02 polypeptides, and because the two catalytic domains are preceded by a predicted hydrophobic N-terminal signal peptide (Fig. 1B), we have renamed the candidate **e**xtracellular (**f**ive prime)-**n**ucleotidase enzymes Efn1 and Efn2, respectively. We will henceforth refer to their genes as *efn1* and *efn2*, respectively (Fig. 1A).

We know the *efn1* and *efn2* genes are induced within 4 h in response to acute phosphate starvation. The phosphate starvation-driven increase in *efn1* mRNA is dampened by a factor of 13 in *pho7*∆ cells vis-à-vis *pho7*^+^ wild-type controls (8), signifying that *efn1* expression, like that of the canonical *PHO* regulon genes, is controlled by transcription factor Pho7. Indeed, the apparent transcription start region of the *efn1* gene is preceded by a TATA-box, upstream of which are four predicted Pho7 DNA-binding sites (8).

The key questions addressed in the present study are as follows. First, are Efn1 and Efn2 proteins indeed extracellular, that is, are they secreted into the medium by phosphate-starved cells? By proteomic analysis of the medium harvested from phosphate-starved fission yeast, we delineate a starvation “secretome” that includes Efn1 and Efn2 (and Pho1) among the most abundant exported proteins. Second, does *S. pombe* secrete a 5'-nucleotidase enzyme activity into the medium during phosphate starvation and, if so, is this activity attributable to Efn1 and Efn2? Bearing in mind that (i) the Pho1 phosphomonoesterase enzyme is overproduced during phosphate starvation, and a minority of that Pho1 is released into the medium rather than being retained on the cell surface (9), and (ii) Pho1 will hydrolyze AMP to adenosine and phosphate (and thus score in a 5'-nucleotidase assay), we detected and characterized a 5'-nucleotidase activity secreted into the medium of phosphate-starved *pho1*∆ *pho4*∆ cells that express no secreted *p*-nitrophenylphosphatase activity. We then affirmed that this extracellular 5'-nucleotidase activity is effaced by the deletion of *efn1* and *efn2*. Thus, the elaboration of 5'-nucleotidase enzymes during phosphate limitation is a newly appreciated feature of fission yeast phosphate homeostasis, with the ability to provide NMP-derived phosphate to phosphate-starved cells.

## RESULTS

### Secretome proteomics of phosphate-starved fission yeast

**FIG 2:**
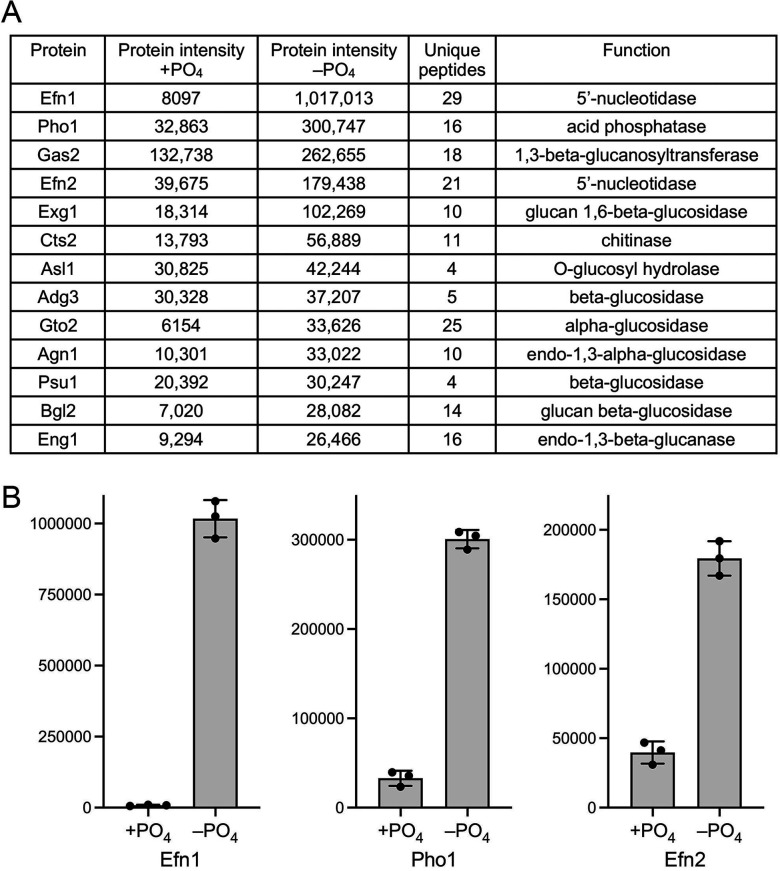
Secretome proteomics of phosphate-starved fission yeast. (**A**) A list of the 13 most abundant secreted proteins detected in the medium of phosphate-starved fission yeast cells (based on a protein intensity value of >25,000 as the average of three biological replicates). (**B**) Pho1, Efn1, and Efn2 are induced and secreted in response to phosphate starvation. Specific protein intensity values obtained from proteomic analysis of the culture medium of fission yeast grown for 12 h in phosphate-replete ePMGT medium or phosphate-free ePMGT(–PO_4_) medium are plotted in bar graph format. The bar heights are average (±SEM) of three replicate cultures.

Twenty-three fission yeast proteins are annotated in Pombase (www.pombase.org/term/GO:0005576) under the term “extracellular region”—in most cases by inference without direct evidence. Here, we conducted experiments to analyze the protein content of the culture medium after the growth of fission yeast for 12 h in a defined synthetic liquid medium (ePMGT; ref. 3) containing or lacking phosphate. Three biological replicate cultures of phosphate-replete and phosphate-starved cells were separated by centrifugation into cell pellet and medium supernatant fractions. The supernatants were passed through 0.22 µm filters, and then concentrated by ultrafiltration with a molecular weight cutoff (MWCO) of 3 kDa. After cysteine reduction and alkylation, the samples were subjected to proteolysis with Lys-C and trypsin. The peptides were analyzed by ultra high performance liquid chromatography (UHPLC)-mass spectrometry, assigned to annotated *S. pombe* proteins, and quantified as protein intensity using Spectronaut software. A list of the 13 most abundant secreted proteins detected in the medium of phosphate-starved fission yeast cells (based on a protein intensity value of >25,000 as the average of three biological replicates) is compiled in Fig. 2A. The list was topped by Efn1, which was increased by 125-fold during the 12 h interval of phosphate starvation vis-à-vis the low basal level present in the medium of phosphate-replete cells (Fig. 2B). Second place in the secretome list was taken by Pho1, which was increased by ninefold as a result of phosphate starvation (Fig. 2B). Efn2, fourth on the list, was induced by approximately fivefold in response to phosphate starvation (Fig. 2B). Whereas Pho1, Efn1, and Efn2 have known or imputed functions in extracellular phosphate mobilization, the 10 other major constituents of the secretome are enzymes involved in cell wall metabolism, many of which were increased by two- to fivefold in response to phosphate starvation (Fig. 2A). Our previous RNA-seq analysis showed that mRNAs encoding two of these cell wall metabolizing enzymes were upregulated in response to phosphate starvation, that is, *exg1* (up eightfold) and *gto2* (up threefold) (3). Experiments presented below address whether the secreted Efn1/2 paralogs are active as 5'-nucleotidases. As a prelude to that inquiry, we first needed to eliminate secreted acid phosphatase as a potentially confounding activity.

### Secreted acid phosphatase activity is effaced by deletion of *pho1* and *pho4*

**FIG 3:**
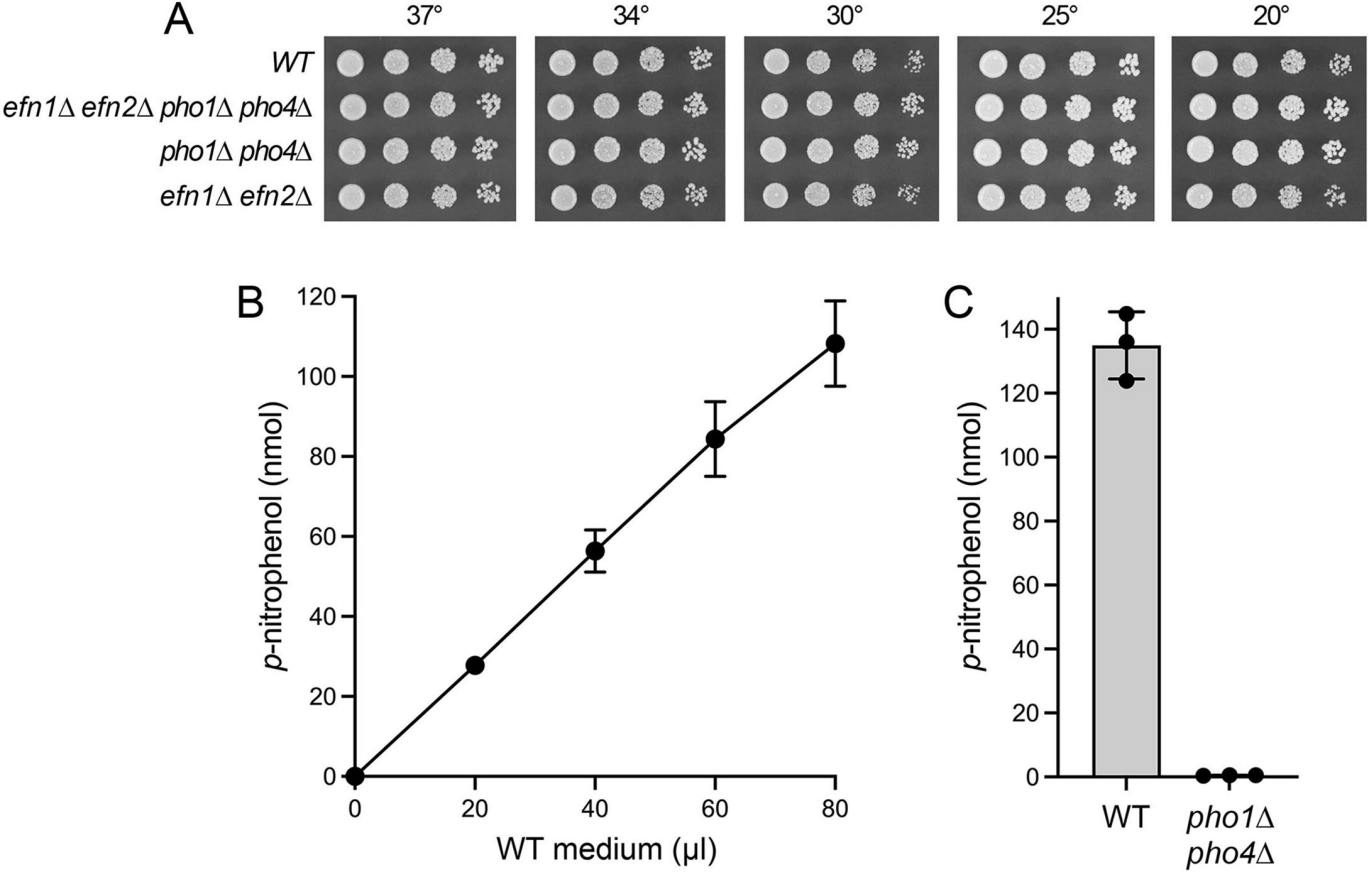
Secretion of acid phosphatase activity during phosphate starvation is effaced by deletion of *pho1* and *pho4*. (**A**) Serial fivefold dilutions of fission yeast strains (as specified on the left) were spot tested for growth on YES agar at the indicated temperatures. (**B**) Reaction mixtures (200 µL) containing 100 mM sodium acetate (pH 4.2), 10 mM *p*-nitrophenylphosphate, and 0, 20, 40, 60, or 80 µL of medium from phosphate-starved wild-type cells were incubated for 5 min at 30°C. The yield of *p*-nitrophenol is plotted as a function of the volume of medium added. The data are averages (±SD) of titration assays using medium from three independent cultures of phosphate-starved cells. (**C**) Reaction mixtures (200 µL) containing 100 mM sodium acetate (pH 4.2), 10 mM *p*-nitrophenylphosphate, and 80 µL of medium from phosphate-starved wild-type or *pho1*∆ *pho4*∆ cells were incubated for 5 min at 30°C. The extents of *p*-nitrophenol release are averages (±SD) of assays using medium from three independent cultures of phosphate-starved wild-type or *pho1*∆ *pho4*∆ cells.

*S. pombe* elaborates two acid phosphatase enzyme paralogs—Pho1 (453-aa) and Pho4 (463-aa)—encoded by the *pho1* and *pho4* genes, respectively (9, 16). Pho1 and Pho4 contain cleavable N-terminal signal peptides and are N-glycosylated in their mature forms (17–20). Pho1 is the predominant cell surface-associated acid phosphatase. Pho1 expression is regulated by phosphate and adenine, whereby transcription of *pho1* is repressed under phosphate-replete and adenine-replete conditions and derepressed by phosphate starvation or adenine starvation, with phosphate status having the greater effect on Pho1 activity (1, 3, 21, 22). Pho4 expression is regulated by thiamine, being repressed under thiamine-replete conditions and derepressed by thiamine starvation (16, 19). As noted above, substantial Pho1 secretion into the medium was evident under phosphate-replete conditions (mean protein intensity 32,863) and was increased by ninefold during phosphate starvation. By contrast, Pho4 was present at a relatively low level in the medium of phosphate-replete cells (mean protein intensity 5,549) and was hardly affected by phosphate starvation (mean protein intensity 6,178). To minimize or eliminate secreted acid phosphatase as confounding factors in our attempts to assay 5’-nucleotidase activity in the medium, we generated a *pho1*∆ *pho4*∆ strain that grew as well as wild-type on YES agar medium (Fig. 3A). Wild-type and *pho1*∆ *pho4*∆ cells were starved for phosphate for 24 h, and the medium supernatants were assayed for acid phosphatase activity via conversion of 10 mM *p*-nitrophenylphosphate to *p*-nitrophenol. The yield of *p*-nitrophenol increased with the amount of wild-type medium added to the reaction mixture (Fig. 3B). Deletion of *pho1* and *pho4* abolished secretion of acid phosphatase activity into the medium of phosphate-starved cells (Fig. 3C).

### Detection of 5'-nucleotidase activity secreted by phosphate-starved *pho1*∆ *pho4*∆ cells

**FIG 4:**
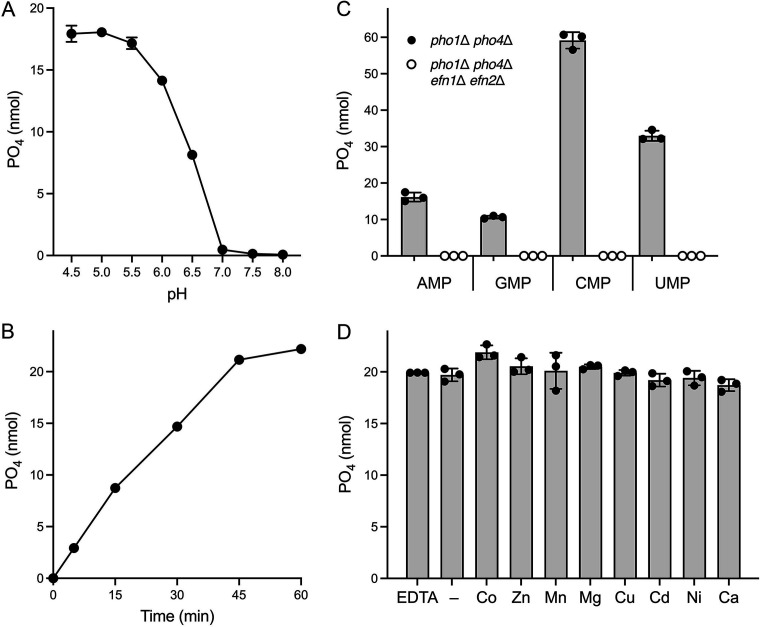
Secretion of 5'-nucleotidase activity during phosphate starvation is effaced by deletion of *efn1* and *efn2*. (**A**) Secreted 5'-nucleotidase activity is optimal at acidic pH. Reaction mixtures (200 µL) containing 50 mM Tris buffer (either Tris-acetate pH 4.5, 5.0, 5.5, 6.0, or 6.5 or Tris-HCl pH 7.0, 7.5, or 8.0), 1 mM AMP, and 25 µL of medium from phosphate-starved *pho1*∆ *pho4*∆ cells were incubated for 30 min at 37°C. Release of free phosphate was determined using the Malachite Green reagent and is plotted as a function of pH. The data are averages (±SD) of three assays using medium from three independent cultures of phosphate-starved *pho1*∆ *pho4*∆ cells. (**B**) Time course. Reaction mixtures (200 µL) containing 50 mM Tris-acetate (pH 5.5), 1 mM AMP, and 25 µL of medium from phosphate-starved *pho1*∆ *pho4*∆ cells were incubated at 37°C. The reactions were quenched after 5, 15, 30, 45, or 60 min by the addition of Malachite Green reagent. The time 0 mixture was quenched prior to adding enzyme. Release of free phosphate is plotted as a function of reaction time. The data are averages (±SD) of assays using medium from three independent cultures of phosphate-starved *pho1*∆ *pho4*∆ cells. (**C**) NMP specificity. Reaction mixtures (200 µL) containing 50 mM Tris-acetate (pH 5.5), 1 mM AMP, GMP, CMP, or UMP, and 25 µL of medium from phosphate-starved *pho1*∆ *pho4*∆ or *pho1*∆ *pho4*∆ *efn1*∆ *efn2*∆ cells were incubated for 30 min at 37°C. Release of free phosphate is plotted. The data are averages (±SD) of three assays using medium from three independent cultures of phosphate-starved *pho1*∆ *pho4*∆ or *pho1*∆ *pho4*∆ *efn1*∆ *efn2*∆ cells. (**D**) Divalent cations. Reaction mixtures (200 µL) containing 50 mM Tris-acetate (pH 5.5), 1 mM CMP, and 8 µL of medium from phosphate-starved *pho1*∆ *pho4*∆ cells, and either no added divalent cation (–) or 1 mM of CoCl_2_, ZnCl_2_, MnCl_2_, MgCl_2_, CuCl_2_, CdSO_4_, NiCl_2_, or CaCl_2_ were incubated for 30 min at 37°C. The EDTA reaction mixture contained no added divalent cation and 8 µL of medium that had been treated with 10 mM EDTA for 10 min prior to its addition to the reaction mixture. Release of free phosphate is plotted. The data are averages (±SD) of three assays using medium from three independent cultures of phosphate-starved *pho1*∆ *pho4*∆ cells.

Medium harvested from cultures of *pho1*∆ *pho4*∆ cells that had been starved for phosphate for 24 h was assayed for 5'-nucleotidase activity. Reaction mixtures containing 1 mM AMP and 50 mM Tris-acetate or Tris-HCl buffer were incubated at 37°C for 30 min, and the formation of inorganic phosphate was quantified colorimetrically using the Malachite Green reagent. 5'-Nucleotidase activity was optimal at acidic pH (between 4.5 and 5.5) and declined sharply as the pH was raised (Fig. 4A). All subsequent 5'-nucleotidase assays were performed in the presence of Tris-acetate buffer, pH 5.5. The extent of AMP hydrolysis to P_i_ increased steadily as a function of incubation time (Fig. 4B).

We compared the ability of medium derived from phosphate-starved *pho1*∆ *pho4*∆ cells to hydrolyze 1 mM AMP, GMP, CMP, and UMP. We observed a hierarchy of phosphate release whereby CMP > UMP > AMP > GMP (Fig. 4C). To query whether the observed 5'-nucleotidase activity derived from Efn1 and Efn2, we constructed an *efn1*∆ *efn2*∆ double mutant and then a *pho1*∆ *pho4*∆ *efn1*∆ *efn2*∆ quadruple mutant, both of which grew as well as wild-type on YES agar medium (Fig. 3A). Assays of medium from cultures of *pho1*∆ *pho4*∆ *efn1*∆ *efn2*∆ cells that had been starved for phosphate for 24 h showed that 5'-nucleotidase activity with AMP, GMP, UMP, and CMP was effaced by deletion of *efn1* and *efn2* (Fig. 4C). We surmise that Efn1 and Efn2 are *bona fide* extracellular 5'-nucleotidase enzymes.

The defining feature of the binuclear metallophosphoesterase enzyme family that includes 5'-nucleotidases is the octahedral coordination of two closely spaced transition metal ions by a conserved constellation of amino acids, comprising two aspartates, three histidines, and an asparagine. His and Asn are “soft” metal ligands via their side chain nitrogen atoms. The catalytic metals engage the scissile phosphate and the water nucleophile. Metallophosphoesterases from disparate sources display distinct divalent cation preferences and, in some instances, have heteronuclear active sites occupied by two different transition metal ions (23–26). The crystal structure of the human 5'-nucleotidase CD73 has two zinc ions in the active site (13). The enzymatic activity of native CD73 was eliminated by extensive dialysis against EDTA to remove endogenous metals; activity was reconstituted by provision of Zn^2+^ or, to a lesser degree, Co^2+^, but not by Ni^2+^, Cu^2+^, Fe^2+^, Mn^2+^, or Mg^2+^ (13). Here, the 5'-nucleotidase activity secreted into the medium of phosphate-starved *pho1*∆ *pho4*∆ cells was evident in the absence of any added divalent cation (Fig. 4A through C). Adding divalent cations Co^2+^, Zn^2+^, Mn^2+^, Mg^2+^, Cu^2+^, Cd^2+^, Ni^2+^, or Ca^2+^ at 1 mM concentration had no effect on the extent of CMP hydrolysis (Fig. 4D), which suggests that the fission yeast 5'-nucleotidases are likely secreted as mature metalloenzymes (*à la* native human CD73). (Note that ePMGT medium contains the following concentrations of divalent cations: 5.2 mM Mg^2+^, 0.1 mM Ca^2+^, 23.7 µM Mn^2+^, 13.9 µM Zn^2+^, 7.4 µM Fe^2+^, 1.6 µM Cu^2+^.) Pre-incubation of the medium of phosphate-starved *pho1*∆ *pho4*∆ cells with 10 mM EDTA for 10 min before assaying CMP hydrolysis did not affect activity (Fig. 4D).

### Secretion of 5'-nucleotidase activity by phosphate-starved *efn1*∆ *pho1*∆ *pho4*∆ and *efn2*∆ *pho1*∆ *pho4*∆ cells

**FIG 5:**
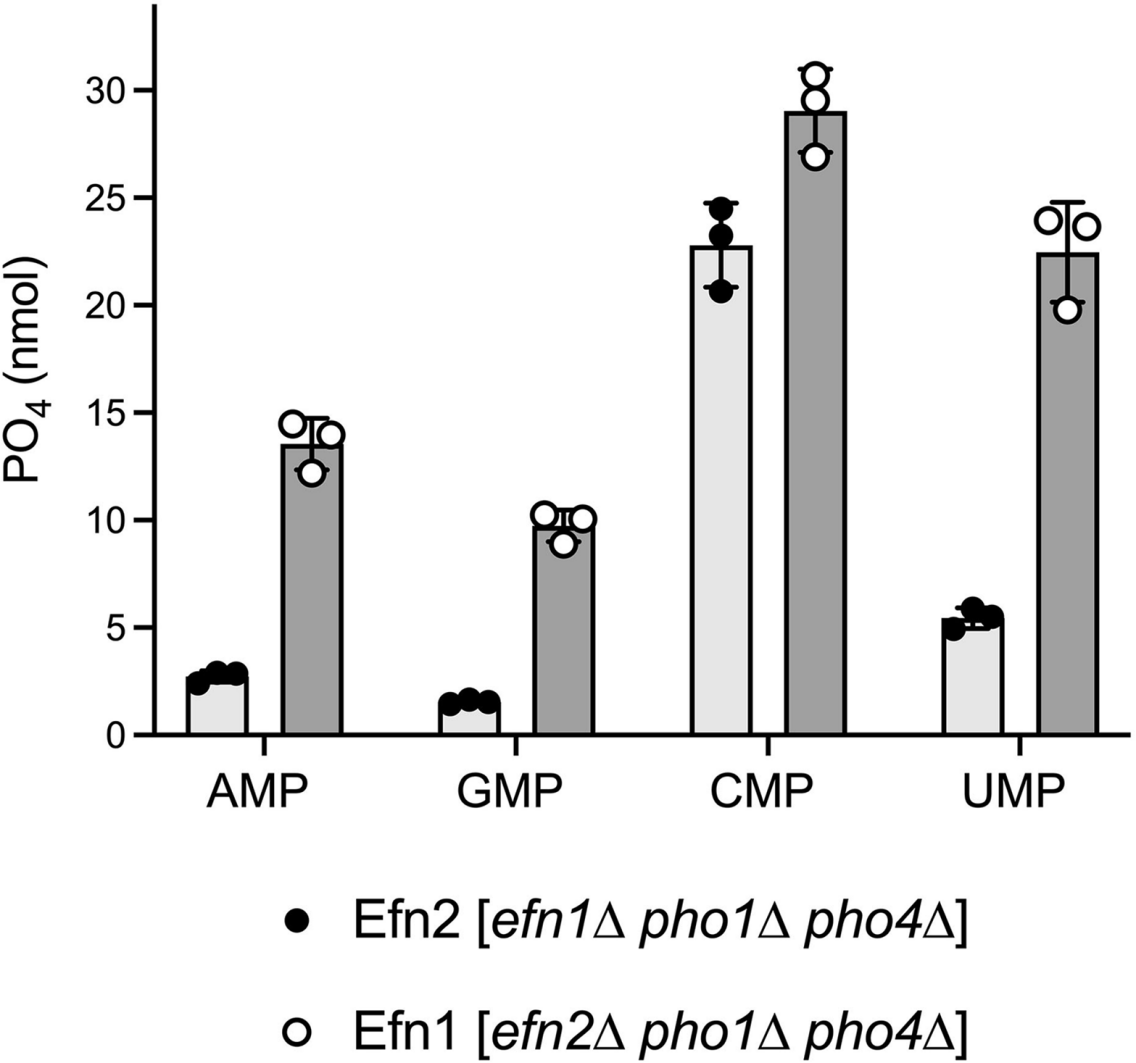
Relative contributions of Efn1 and Efn2 to secreted 5'-nucleotidase activity. Reaction mixtures (200 µL) containing 50 mM Tris-acetate (pH 5.5), 1 mM AMP, GMP, CMP, or UMP, and 25 µL of medium from phosphate-starved *efn1*∆ *pho1*∆ *pho4*∆ or *efn2*∆ *pho1*∆ *pho4*∆ cells were incubated for 30 min at 37°C. Release of free phosphate is plotted. The data are averages (±SD) of three assays using medium from three independent cultures of phosphate-starved *efn1*∆ *pho1*∆ *pho4*∆ or *efn2*∆ *pho1*∆ *pho4*∆ cells.

To gauge the relative contributions of Efn1 and Efn2 to the observed 5'-nucleotidase activity, we individually deleted the *efn1* and *efn2* genes in a *pho1*∆ *pho4*∆ background, subjected the resulting *efn1*∆ *pho1*∆ *pho4*∆ and *efn2*∆ *pho1*∆ *pho4*∆ strains to 24 h of phosphate starvation, and harvested the culture medium for 5'-nucleotidase assays with all four rNMP substrates. The premise here is that the activity detected in *efn1*∆ *pho1*∆ *pho4*∆ culture medium is assigned to Efn2, whereas Efn1 is responsible for any activity detected in *efn2*∆ *pho1*∆ *pho4*∆ culture medium. Figure 5 depicts the extent of NMP hydrolysis by 25 µL of culture medium from phosphate-starved *efn1*∆ and *efn2*∆ cells, the same volume of medium from *efn1*^+^ *efn2*^+^ cells assayed in Fig. 4C. Note that the sum of the activities in the media of the single *efn* null mutants agrees with that of the *efn1*^+^ *efn2*^+^ strain for each NMP substrate. It is apparent that Efn1 contributes a greater share of secreted 5'-nucleotidase activity against AMP, GMP, and UMP compared to Efn2. The Efn1:Efn2 activity ratio is 5.0 for AMP, 6.3 for GMP, and 4.1 for UMP (Fig. 5). These results are in accord with the relative abundance of the Efn1 and Efn2 polypeptides in the medium of phosphate-starved fission yeast cultures (Fig. 2A). However, the Efn1:Efn2 activity ratio for CMP hydrolysis is 1.3, suggesting that Efn2 displays a greater selectivity for CMP than does Efn1.

### Absence of acid phosphatases and 5'-nucleotidases does not affect survival during phosphate starvation

Fission yeast cells adapt to chronic phosphate starvation by entering a state of G0 quiescence, initially fully reversible upon replenishing phosphate after 2 days of starvation (2). Time-resolved analyses of transcriptome changes revealed coherent perturbations of gene expression whereby the mRNAs encoding the cellular machineries for ribosome biogenesis, tRNA biogenesis, and protein translation are globally downregulated, whereas those for autophagy and phosphate mobilization are upregulated. At the proteome level, phosphate starvation results in depletion of ribosome assembly factors, 60S and 40S proteins, tRNA-modifying enzymes, and translation factors. Wild-type fission yeast cells progressively lose viability in the interval between 2 days (100% viable), 14 days (44% viable), and 28 days (4% viable) of phosphate starvation (2). Previously, we identified Maf1, a negative regulator of RNA polymerase III transcription, as a key determinant of the chronological lifespan of phosphate-starved fission yeast, insofar as (i) *maf1* mRNA and Maf1 protein are upregulated during a 48 h period of phosphate starvation, and (ii) *maf1*∆ cells undergo accelerated demise between 1 and 2 days of phosphate starvation culminating in less than 1% survival after 4 days (2). The 24–48 h temporal window during which phosphate-starved *maf1*∆ cells begin to expire is associated with overproduction of tRNA and the accumulation of polyadenylated tRNAs, intron-containing pre-tRNAs, and unspliced tRNA fragments (2). We proposed that Maf1 prolongs chronological lifespan during phosphate starvation by repressing Pol III and preventing a death pathway associated with aberrant tRNA metabolism. Autophagy also contributes to the survival of phosphate-starved cells, whereby deletion of the gene encoding the serine/threonine kinase Atg1 resulted in accelerated death, with 50% survival after 2 days of phosphate deprivation and less than 6% survival after 7 days (8).

Transcription factor Pho7, which is a driver of the expression of phosphate acquisition genes *pho1*, *pho84*, *tgp1*, and *efn1* during phosphate starvation, also impacts survival, albeit to a lesser degree than Maf1. *pho7*∆ cells display reduced viability after 4 and 7 days of phosphate starvation (22% and 14% survival, respectively) (8). It was suggested that Pho7 promotes the expression of gene(s) that extend the chronological lifespan of phosphate-starved cells.

**FIG 6:**
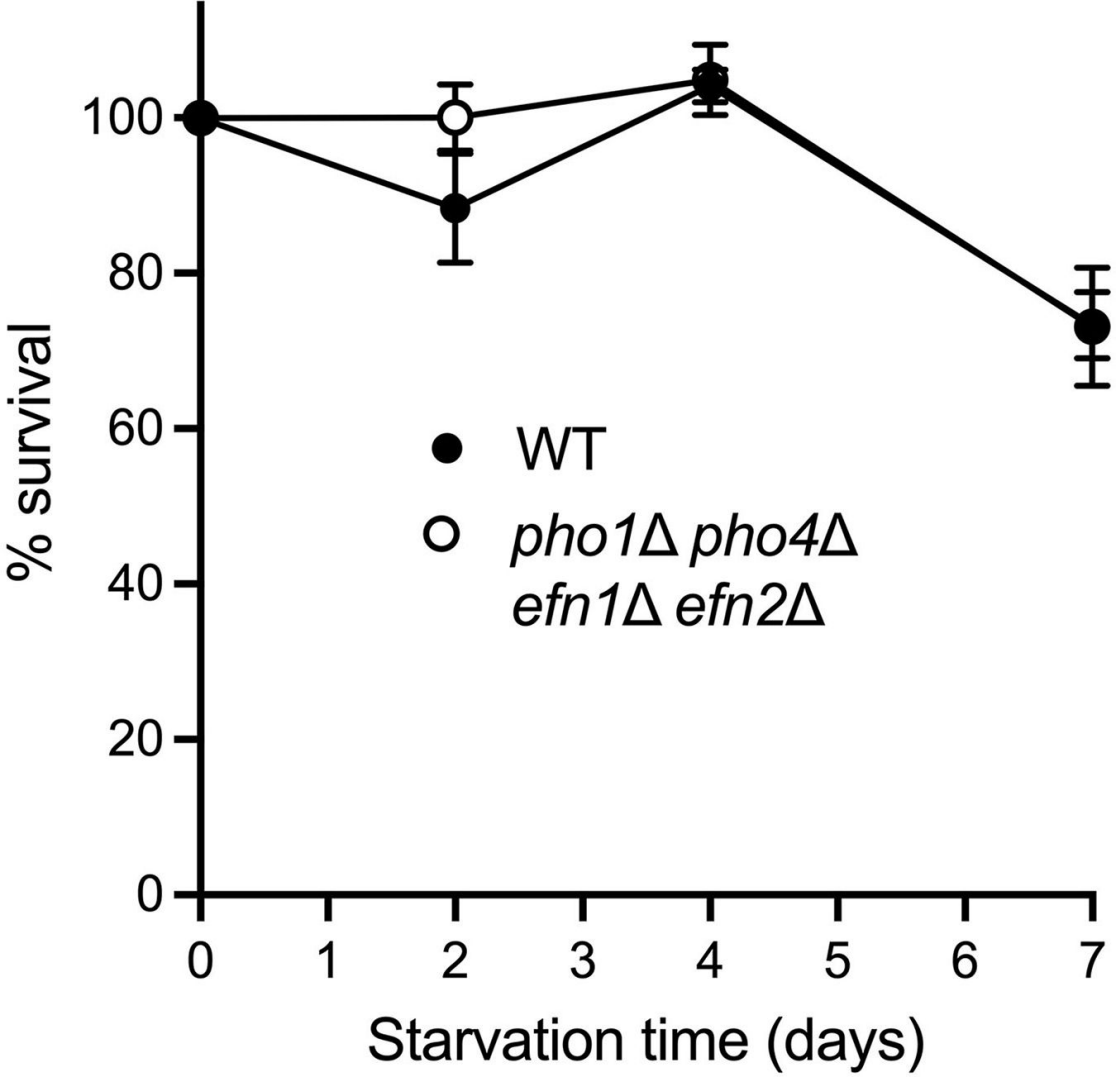
Deletion of *pho1*, *pho4*, *efn1*, and *efn2* does not impact the viability of fission yeast cells during 7 days of phosphate starvation. Independent replicate cultures of wild-type and *pho1*∆ *pho4*∆ *efn1*∆ *efn2*∆ fission yeast cells were grown at 30°C in ePMGT medium to *A*_600_ of 0.4–0.7. The cells were harvested, washed with water, and adjusted to *A*_600_ of 0.03 in ePMGT medium without phosphate. An aliquot was withdrawn from each replicate before transfer (0 time) and after 2, 4, and 7 days of phosphate starvation. Cell number was determined with a hemacytometer. The cells were adjusted to 100,000 cells/mL and aliquots of serial dilutions (~1,000 and 100 cells) were plated on ePMGT agar medium and incubated for 3 days at 30°C to determine the number of colony-forming units (CFUs). Viable cell counts were normalized to the time 0 control (100%). Percent survival (average of at least three biological replicate experiments ± SEM) is plotted as a function of starvation time.

Here, we interrogated the effect of ablating the expression of cell surface-associated and secreted acid phosphatase and 5'-nucleotidase enzymes on the lifespan of phosphate-starved fission yeast. To address this point, we compared the survival of wild-type and *pho1*∆ *pho4*∆ *efn1*∆ *efn2*∆ cells that were subjected to phosphate starvation for up to 7 days, then allowed to recover growth on phosphate-replete medium. Aliquots of cells were collected prior to (time 0) and 2, 4, and 7 days after transfer from ePMGT to ePMGT–PO_4_ and counted with a hemacytometer. Serial dilutions were plated on ePMGT agar medium and incubated at 30°C. Viable colony counts were normalized to the time 0 control, and percent survival was plotted as a function of starvation time (Fig. 6). Wild-type and *pho1*∆ *pho4*∆ *efn1*∆ *efn2*∆ cells retained full viability after 2 days of starvation before viability declined to 73% survival after 7 days of starvation. We conclude that Pho1 acid phosphatase and extracellular 5'-nucleotidase activities, which are induced during acute phosphate starvation, are not important for survival during chronic phosphate starvation. It is likely that the lifespan-shortening effects of ablating Pho7 reflect its role in promoting the expression of several hundred other fission yeast genes (8).

### Efn1 and Efn2 enable the use of extracellular CMP as a source of inorganic phosphate during phosphate starvation

**FIG 7:**
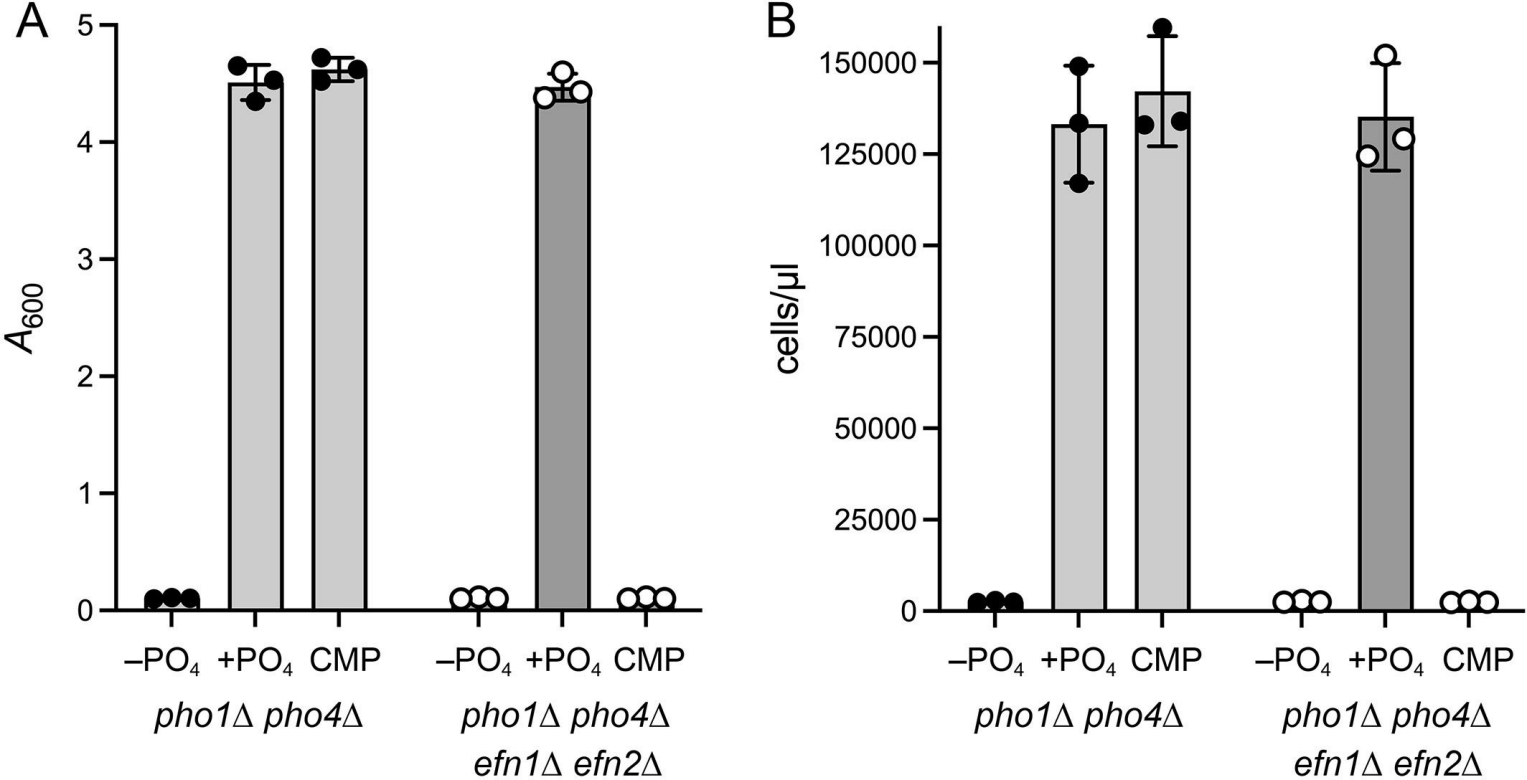
Secreted 5'-nucleotidase activity enables utilization of extracellular CMP as a source of phosphate during phosphate starvation. *pho1*Δ *pho4*Δ and *pho1*Δ *pho4*Δ *efn1*Δ *efn2*Δ cells were grown at 30°C in ePMGT medium to *A*_600_ of 0.6–0.8. The cells were harvested, washed with water, adjusted to *A*_600_ of 0.01 in either ePMGT(–PO_4_), ePMGT(–PO_4_) supplemented with 1 mM phosphate, or ePMGT(–PO_4_) supplemented with 1 mM CMP and grown for 24 h at 30°C, after which the *A*_600_ of the cultures was measured (A) and cell counts were determined with a hemocytometer (B). The data in the bar graphs are averages (±SD) of three experiments using independent replicate cultures of *pho1*Δ *pho4*Δ and *pho1*Δ *pho4*Δ *efn1*Δ *efn2*Δ cells.

Fission yeast *pho1*∆ *pho4*∆ cells and *pho1*∆ *pho4*∆ *efn1*∆ *efn2*∆ cells that were growing logarithmically in ePMGT(+PO_4_) medium were harvested, washed with water, and resuspended at an *A*_600_ of 0.01 in either (i) ePMGT(–PO_4_) medium, (ii) ePMGT(–PO_4_) supplemented with 1 mM phosphate, or (iii) ePMGT(–PO_4_) supplemented with 1 mM CMP. The cultures were incubated for 24 h at 30°C, after which growth was assessed by *A*_600_ and counting the cells microscopically using a hemacytometer. As expected, the growth of both yeast strains was curtailed in the absence of phosphate and was rescued by inclusion of 1 mM phosphate in the culture medium (Fig. 7). The instructive findings were that 1 mM CMP was equally effective in sustaining the growth of *pho1*∆ *pho4*∆ cells during a 24 h period of phosphate starvation, whereas *pho1*∆ *pho4*∆ *efn1*∆ *efn2*∆ cells were unable to use CMP as a phosphate source (Fig. 7).

## DISCUSSION

Here, we identified two paralogous extracellular 5'-nucleotidase enzymes—Efn1 and Efn2—and the acid phosphatase Pho1 as major constituents of the secretome of phosphate-starved fission yeast cells. Efn1 and Efn2 are members of the binuclear metallophosphoesterase enzyme superfamily and are encoded by genes that are strongly upregulated during acute and chronic phosphate deprivation (2). We demonstrate that Efn1 and Efn2 account for all the 5'-nucleotidase activity secreted by phosphate-starved fission yeast cells that lack acid phosphatase Pho1 and its paralog Pho4. Our characterization of Efn1/Efn2 activities revealed the following features: (i) an acid pH optimum; (ii) lack of requirement for an exogenous divalent cation, implying that they are secreted as mature metalloenzymes; and (iii) hydrolysis of all four standard ribonucleoside monophosphates at 1 mM concentration.

By singly deleting the *efn1* and *efn2* genes in the *pho1*∆ *pho4*∆ background, we find that Efn1 contributes the greater share of secreted 5'-nucleotidase activity against AMP, GMP, and UMP vis-à-vis Efn2. Efn1 and Efn2 display their highest activity with 1 mM CMP, and it appears that Efn2 is especially selective for CMP (at least in the context of conditioned culture medium from phosphate-starved cells). Whereas many studies of 5'-nucleotidase enzymes from diverse sources have focused on adenosine nucleotide substrates and analogs (12, 14), there is no *a priori* reason to think that these enzymes need to be strictly specific for AMP. Indeed, steady-state kinetic analyses of soluble secreted CD73 (sCD73) expressed in human cells and purified from culture medium revealed similar *k*_cat_ values for AMP (187 s^−1^), GMP (132 s^−1^), CMP (202 s^−1^), and UMP (289 s^−1^) at saturating NMP concentrations (13). sCD73 displayed eightfold higher affinity for the purine NMPs (*K*_m_ of 3.8 µM and 6.5 µM for AMP and GMP, respectively) versus the pyrimidine NMPs (*K*_m_ of 32 µM and 56 µM for UMP and CMP, respectively) (13). An alternative method of preparing catalytically active recombinant CD73 by denaturation/refolding of CD73 from insoluble bacterial inclusion bodies affirmed that CD73 hydrolyzed all four rNMPs at 1 mM concentration (15). Crystal structures of CD73 in complex with AMP, GMP, CMP, and UMP highlighted the exact superposition of their ribose and phosphate moieties and coplanar π-stacking of the respective nucleobases between two phenylalanine side chains conserved among 5'-nucleotidases (15; also see below).

**FIG 8:**
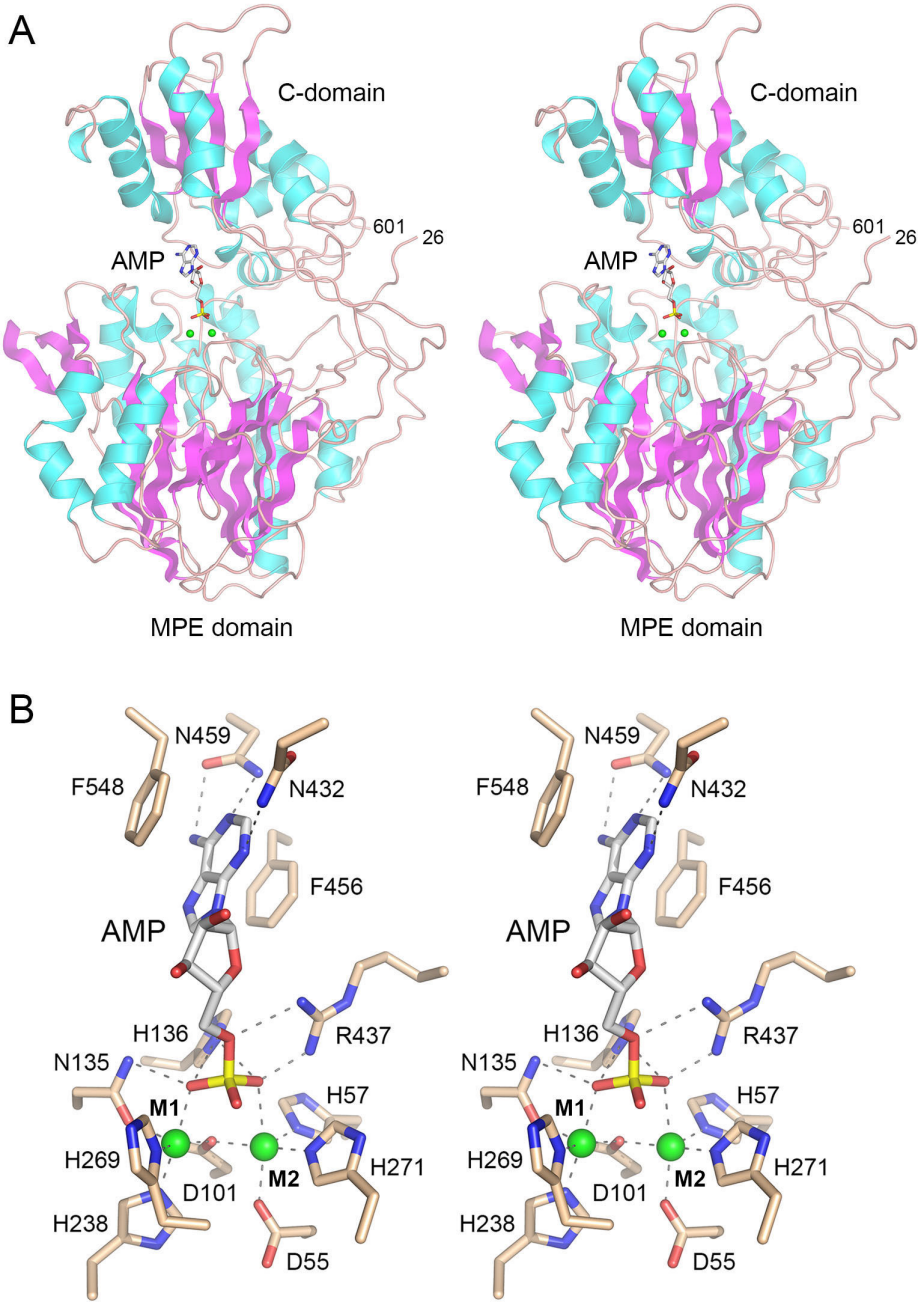
AlphaFold 3 model of Efn1 in complex with two divalent metal ions and AMP. (**A**) Stereo view of the Efn1 model (aa 26–601, lacking the N-terminal signal peptide), depicted as a cartoon trace with cyan α-helices and magenta β-strands. Two cobalt ions (green spheres) demarcate the active site within the N-terminal metallophosphoesterase (MPE) domain. The adenosine moiety of AMP is engaged by the C-terminal domain. (**B**) Stereo view of the active site highlighting predicted atomic contacts to the M1 and M2 metal ions (green spheres) and the AMP phosphate oxygens. The nucleobase fits into a π-sandwich between two phenylalanine side chains.

We exploited the AlphaFold 3 server (27) to generate a model of Efn1 in complex with AMP and two divalent metal ions, arbitrarily chosen as cobalt. The predicted Efn1 tertiary structure (shown in stereo view in Fig. 8A) adopts a “closed” 5'-nucleotidase conformation (14, 28) in which the N-terminal metallophosphoesterase domain and the C-terminal nucleoside-binding domain simultaneously engage the AMP substrate and the enzyme is poised for catalysis. A stereo view of the predicted Efn1 active site is shown in Fig. 8B. The M1 catalytic metal ion is engaged by Asp101-Οδ, Asn135-Οδ, His238-Nε, His269-Nδ, and a phosphate oxygen. The M2 catalytic metal is coordinated by Asp55-Oδ, His57-Nε, Asp101-Οδ, His271-Nε, and a phosphate oxygen. A putative water nucleophile (missing from the AlphaFold model) is expected to jointly occupy the sixth position of the octahedral M1 and M2 complexes, stabilized as a hydroxide ion and poised below the AMP phosphate in apical orientation to the adenosine O5' leaving group. Contacts from the catalytic metals and Asn135-Nδ, His136-Nε, and Arg437 to the three nonbridging AMP phosphate oxygens are predicted to stabilize the extra negative charge developed in a pentacoordinate phosphorane transition state. Hydrogen bonds from Arg437 and/or His136-Nε to the O5' leaving group may aid general acid catalysis of leaving group expulsion. The adenine nucleobase is sandwiched in a π-stack between Phe456 and Phe548. Asn459 makes bidentate hydrogen bonds from Oδ to adenine-N6 and from Nδ to adenine-N1 (Fig. 8B). A simple rotamer flip of Asn459 could accommodate hydrogen bonding to guanine-O6 and guanine-N1. Asn432-Nδ makes a hydrogen bond to adenine-N3 in the AlphaFold3 model (Fig. 8B). In this rotamer, Asn432-Oδ could engage in an additional hydrogen bond to the exocyclic 2-amino group of a guanine base. Direct or water-mediated hydrogen bonding of these asparagine residues to pyrimidine nucleobases is also feasible. In sum, the Efn1 structure predicted by AlphaFold 3 appears to be on pathway and is informative with respect to catalysis and NMP promiscuity.

Expression of the *efn1* and *efn2* genes is rapidly and strongly upregulated as part of an acute phosphate starvation response that includes the classic *PHO* regulon genes *pho1*, *pho84*, and *tgp1* (1, 3). Collectively, the elaboration of cell surface and secreted acid phosphatase and secreted 5'-nucleotidase activities will mobilize phosphate from various organic phosphate-containing compounds in the environment and thereby, at least in the short term, help blunt the effects of a sudden complete absence of extracellular inorganic phosphate (as imposed during laboratory experiments) or, more plausibly in the wild, allow fission yeast cells to grow in a milieu in which inorganic phosphate is present at scant concentrations. Our demonstration that secretion of Efn1/2 enables phosphate-starved fission yeast to thrive by using extracellular CMP as a source of inorganic phosphate highlights their pro-adaptive function. The enzymatically mobilized inorganic phosphate will be taken up by plasma membrane inorganic phosphate transporters Pho84, Pho841, and Pho842 that are also induced during acute phosphate starvation (3). Whereas the acute starvation response is focused on acquiring phosphate, the chronic starvation response is geared toward establishing a quiescent state and prolonging chronological lifespan, in anticipation of a return to growth when phosphate is replenished (3, 8). The chronic adaptations include upregulation of autophagy genes and downregulation of the cellular machineries for ribosome production and translation (3, 8). The present study shows that ablation of the acid phosphatase and 5'-nucleotidase inductions does not shorten the survival of fission yeast during a 7-day interval of complete phosphate starvation.

## MATERIALS AND METHODS

### Phosphate starvation

Three independent fission yeast cultures were grown at 30°C in ePMGT(+PO_4_) medium at 30°C. When the cultures reached mid-log phase (*A*_600_ of ~0.5; 1 *A*_600_ = 2 × 10^7^ cells), the cells were harvested by centrifugation, washed with water, and resuspended in either ePMGT(+PO_4_) to attain *A*_600_ of ~0.1 or in ePMGT(–PO_4_) to attain *A*_600_ of ~0.12. The six cultures (three replicates each of phosphate-replete and phosphate-starved cells) were incubated at 30°C for 12 h. The phosphate-replete cultures were diluted with ePMGT(+PO_4_) as needed during the 12 h incubation so as to not exceed *A*_600_ of 0.8. The phosphate-starved cultures were arrested at *A*_600_ of 0.65–0.69 after 12 h. At the 12 h timepoint, 14 *A*_600_ units of cells from each culture (2.8 × 10^8^ cells) were harvested by centrifugation at 4°C. The supernatants (~20–22 mL) were filtered through a 0.22 µm Millex-MP filter unit (Millipore, Cat# SLMP025SS). Filtered supernatants were collected in two separate 15 mL tubes (~10 mL per tube), then frozen immediately in liquid nitrogen, and kept at −80°C pending proteomic analysis.

### Proteomics analysis: proteolysis

Thawed supernatants were concentrated by centrifugal ultrafiltration (Amicon Ultra centrifugal filters; 3 kDa MWCO) to a final volume of 100 µL and transferred to a new Eppendorf tube. The filters were rinsed with 8 M urea, 50 mM EPPS {3-[4-(2-hydroxyethyl)piperazin-1-yl]propane-1-sulfonic acid} pH 8.5, and the rinse was added to the ultrafiltered concentrate. After protein quantification using the Pierce bicinchoninic acid assay (Thermo Fisher), proteins were reduced by treatment with 500 mM Tris (2-carboxyethyl) phosphine (5 mM final concentration) for 30 min at 25°C with shaking (1,000 rpm) on a Thermomixer (Thermo Fisher). Free cysteine residues were alkylated by treatment with 500 mM iodoacetamide (10 mM final concentration) for 30 min in the dark at room temperature. Subsequently, 1 M DL-dithiothreitol (5 mM final concentration) was added for 15 min at room temperature. The samples were digested with lys-C (Wako) at a 1:100 enzyme:protein ratio for 1 h at 25°C with shaking (1,000 rpm), then diluted with 50 mM ammonium bicarbonate and digested with trypsin (Promega) at a 1:100 enzyme:protein ratio at 37°C overnight with shaking (1150 rpm). After digestion, the peptides were acidified to pH < 3 by adding 50% trifluoroacetic acid (TFA) and then desalted using C18 Sep-Pak cartridges (Waters, Milford, MA, USA) as follows. Cartridges were conditioned by sequential addition of (i) methanol, (ii) 70% acetonitrile (ACN)/0.1% TFA, and (iii) 5% ACN/0.1% TFA twice. Following conditioning, the acidified peptide digest was loaded onto the cartridge under vacuum. The stationary phase was washed twice with 5% ACN/0.1% formic acid (FA). Finally, peptides were eluted from the cartridge with 70% ACN/0.1% FA. Eluted peptides were dried under vacuum, reconstituted in water/0.1% FA, and sonicated in a water bath sonicator. Peptide yield was quantified using a NanoDrop (Thermo Fisher).

### Proteomics analysis: mass spectrometry

Peptides were separated on an IonOpticks Aurora C18 UHPLC column (25 cm × 75 µm; 1.7 µm particle size) composed of C18 stationary phase (IonOpticks Aurora 3 1801220) using a gradient from 2% to 35% buffer B over 90 min and then to 95% buffer B for 7 min (Buffer A: 0.1% FA in HPLC grade water; Buffer B: 99.9% ACN, 0.1% FA) with a flow rate of 300 nL/min using a NanoElute2 system (Bruker). MS data were acquired on a timsTOF HT mass spectrometer (Bruker) with a Captive Spray source (Bruker) using a data-independent acquisition PASEF method (dia-PASEF). The mass range was set from 100 to 1700 *m*/*z*, and the ion mobility range was set from 0.60 V⋅s/cm^2^ (collision energy 20 eV) to 1.6 V⋅s/cm^2^ (collision energy 59 eV) with a ramp time of 100 ms and an accumulation time of 100 ms. Dia-PASEF settings included a mass range 400.0 Da to 1201.0 Da, a mobility range 0.60–1.60, and a cycle time estimate of 1.80 s. The dia-PASEF windows were set with a mass width of 26.00 Da, mass overlap 1.00 Da, and 32 mass steps per cycle.

### Proteomics data analysis

Raw data files were processed using Spectronaut version 18.6 (Biognosys) and searched with the PULSAR search engine with a UniProt *Schizosaccharomyces pombe* (strain 972/ATCC 24843) protein database downloaded on 9 February 2024 (5,137 entries). Cysteine carbamidomethylation was specified as fixed modifications, while methionine oxidation, acetylation of the protein N-terminus, and deamidation (NQ) were set as variable modifications. A maximum of two trypsin missed cleavages were permitted. Searches used a reversed sequence decoy strategy to control peptide false discovery rate (FDR) and 1% FDR was set as a threshold for identification. Unpaired *t*-test was used to calculate *P*-value in differential analysis.

### *S. pombe* gene deletions

The uracil prototrophic *pho1*∆ (*pho1::ura4*^+^) and nourseothricin-resistant *pho4*∆ (*pho4::natMX*) strains were described previously (29). To generate *efn1*∆ and *efn2*∆ deletions, we used PCR and standard cloning methods to first construct plasmids in which the *efn1* gene from nucleotides −11 to +1,779 (relative to the translation start codon +1) and the *efn2* gene from nucleotides +1 to +1,806 were replaced by *hygMX* and *kanMX* antibiotic resistance cassettes, respectively. The disruption cassettes, in which the resistance marker is flanked by 500–650 bp segments of upstream and downstream gene-specific chromosomal DNA, were excised from the plasmids and transfected into diploid *S. pombe* cells. Antibiotic-resistant transformants were selected and analyzed by Southern blotting to confirm correct integration at one of the *efn1* or *efn2* loci, thereby effectively deleting the first 593 amino acids of Efn1 or the entire 601-aa Efn2 protein, respectively. Hygromycin-resistant *efn1*∆ or G418-resistant *efn2*∆ haploids were isolated after sporulation of the respective heterozygous diploids. Standard fission yeast genetic methods (30, 31) were used to generate haploid strains harboring deletions in two or more differently marked genes. In brief, pairs of haploids with null mutations, for example, *pho1*∆ (*pho1::ura4*^+^) and *pho4*∆ (*pho4::natMX*) or hygromycin-resistant *efn1*∆ (*efn1::hygMX*) and *efn2*∆ (*efn2::kanMX*) were mixed on malt agar to allow mating and sporulation and then the mixture was subjected to random spore analysis. Spores were plated to YES agar and on media selective for marked mutant alleles and incubated at 30°C. Viable haploid progeny (*n* = 400–500) were screened by replica-plating for the presence of the second marked gene. We thereby recovered *pho1*∆ *pho4*∆ and *efn1*∆ *efn2*∆ double-deletion strains. Triple deletants *pho1*∆ *pho4*∆ *efn1*∆ and *pho1*∆ *pho4*∆ *efn2*∆ and quadruple knockout *pho1*∆ *pho4*∆ *efn1*∆ *efn2*∆ were recovered after crossing the *pho1*∆ *pho4*∆ strain to *efn1*∆, *efn2*∆, and *efn1*∆ *efn2*∆ strains, respectively, and sequentially replica-plating random haploid progeny from YES to selective media. Because the *efn1* and *pho1* genes are closely linked on chromosome II (20 kbp apart), more than 2,000 viable spores were screened for the marked *pho1*∆ and *efn1*∆ genes in order to recover *pho1*∆ *pho4*∆ *efn1*∆ and *pho1*∆ *pho4*∆ *efn1*∆ *efn2*∆ progeny. Genotypes of the compound mutants were confirmed by diagnostic PCR.

### Spot tests of fission yeast growth

Cultures of *S. pombe* strains were grown in liquid YES (yeast extract with supplement) medium until *A*_600_ reached 0.3–0.5. The cultures were adjusted to an *A*_600_ of 0.1 and aliquots (3 µL) of serial fivefold dilutions were spotted to YES agar. The plates were photographed after incubation for 2 days at 30°C and 34°C, 2.5 days at 37°C, 4 days at 25°C, and 6 days at 20°C.

### Secreted acid phosphatase activity

Three independent cultures of wild-type and *pho1*∆ *pho4*∆ cells were grown at 30°C in ePMGT medium to *A*_600_ of 0.6–0.8. The cells were harvested, washed with water, then adjusted to *A*_600_ of 0.2 in ePMGT(–PO_4_) medium, and incubated at 30°C for 24 h. The cultures were centrifuged to separate cell and medium fractions. Acid phosphatase activity in the medium was assayed as follows. Reaction mixtures (200 µL) containing 100 mM sodium acetate (pH 4.2), 10 mM *p*-nitrophenylphosphate, and aliquots of medium as specified in Fig. 3 legend were incubated for 5 min at 30°C. The reactions were quenched by the addition of 1 mL of 1 M sodium carbonate and the absorbance at 410 nm was measured. The extent of formation of *p*-nitrophenol was determined by interpolation of the *A*_410_ values to a *p*-nitrophenol standard curve.

### Secreted 5'-nucleotidase activity

Three independent cultures of *pho1*∆ *pho4*∆ and *pho1*∆ *pho4*∆ *efn1*∆ *efn2*∆ cells were grown at 30°C in ePMGT medium to *A*_600_ of 0.6–0.8. The cells were harvested, washed with water, then adjusted to *A*_600_ of 0.2 in ePMGT(–PO_4_) medium, and incubated at 30°C for 24 h. The cultures were centrifuged to separate cell and medium fractions. 5’-nucleotidase activity in the medium was assayed as follows. Reaction mixture (200 µL) containing 50 mM Tris buffer (pH as specified in the figure legends), 1 mM NMP, and aliquots of medium as specified in the figure legends were incubated at 37°C. The reactions were quenched at the times specified by the addition of 1 mL of Malachite Green reagent (BIOMOL Green, Enzo Life Sciences) and the absorbance at 620 nm was measured after 20 min incubation at room temperature. The extent of released free phosphate was determined by interpolation of the *A*_620_ values to a phosphate standard curve.

## Data Availability

The mass spectrometry proteomics data have been deposited to the ProteomeXchange Consortium via the PRIDE partner repository with the data set identifier PXD056330.
